# A Novel Computational Biomechanics Framework to Model Vascular Mechanopropagation in Deep Bone Marrow

**DOI:** 10.1002/adhm.202201830

**Published:** 2023-01-08

**Authors:** Yunduo Charles Zhao, Yingqi Zhang, Fengtao Jiang, Chi Wu, Boyang Wan, Ruhma Syeda, Qing Li, Bo Shen, Lining Arnold Ju

**Affiliations:** ^1^ School of Biomedical Engineering The University of Sydney 2008 New South Wales Darlington Australia; ^2^ Charles Perkins Centre The University of Sydney 2006 New South Wales Camperdown Australia; ^3^ The University of Sydney Nano Institute (Sydney Nano) The University of Sydney 2006 New South Wales Camperdown Australia; ^4^ School of Aerospace, Mechanical and Mechatronic Engineering The University of Sydney 2008 New South Wales Darlington Australia; ^5^ Department of Neuroscience University of Texas Southwestern Medical Center 75235 TX Dallas USA; ^6^ National Institute of Biological Science Zhongguancun Life Science Park 102206 Beijing China; ^7^ Tsinghua Institute of Multidisciplinary Biomedical Research Tsinghua University 102206 Beijing China

**Keywords:** mechanobiology, stem cell niche, finite elements analysis, bone marrow, osteogenesis, arteriole, sinusoid

## Abstract

The mechanical stimuli generated by body exercise can be transmitted from cortical bone into the deep bone marrow (mechanopropagation). Excitingly, a mechanosensitive perivascular stem cell niche is recently identified within the bone marrow for osteogenesis and lymphopoiesis. Although it is long known that they are maintained by exercise‐induced mechanical stimulation, the mechanopropagation from compact bone to deep bone marrow vasculature remains elusive of this fundamental mechanobiology field. No experimental system is available yet to directly understand such exercise‐induced mechanopropagation at the bone‐vessel interface. To this end, taking advantage of the revolutionary in vivo 3D deep bone imaging, an integrated computational biomechanics framework to quantitatively evaluate the mechanopropagation capabilities for bone marrow arterioles, arteries, and sinusoids is devised. As a highlight, the 3D geometries of blood vessels are smoothly reconstructed in the presence of vessel wall thickness and intravascular pulse pressure. By implementing the 5‐parameter Mooney–Rivlin model that simulates the hyperelastic vessel properties, finite element analysis to thoroughly investigate the mechanical effects of exercise‐induced intravascular vibratory stretching on bone marrow vasculature is performed. In addition, the blood pressure and cortical bone bending effects on vascular mechanoproperties are examined. For the first time, movement‐induced mechanopropagation from the hard cortical bone to the soft vasculature in the bone marrow is numerically simulated. It is concluded that arterioles and arteries are much more efficient in propagating mechanical force than sinusoids due to their stiffness. In the future, this *in‐silico* approach can be combined with other clinical imaging modalities for subject/patient‐specific vascular reconstruction and biomechanical analysis, providing large‐scale phenotypic data for personalized mechanobiology discovery.

## Graphical abstract

The mechanical stimuli generated by body exercise can be transmitted from cortical bone into the deep bone marrow. We devise a computational framework that reconstructs *in‐vivo* 3D deep bone marrow vessels to quantify the mechanopropagation capabilities for arterioles, arteries, and sinusoids. In the future, this framework can combine with other clinical imaging modalities for personalized vascular reconstruction and mechanobiology analyses.

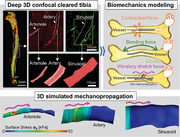

## 1 Introduction

Bone marrow is known to be mechanosensitive.^[^ ^1^ ^]^ Peri‐arteriolar Osteolectin‐expressing (Oln^+^) stromal cells in the bone marrow are critical resources for bone regeneration (osteogenesis) and immune function (lymphopoiesis).^[^ ^2^ ^]^ Nevertheless, it is elusive how bone marrow stem cells sense and respond to their mechanical microenvironment thereafter regulate their differentiation fates. Advancement of this mechanobiology is significant for addressing health issues and diseases with respect to cancer (e.g., osteosarcoma and leukemia),^[^ ^3^ ^]^ aging (e.g., osteoarthritis),^[^ ^4^, ^5^ ^]^ and even spaceflight travel (e.g., osteoporosis)^[^ ^6^ ^]^ in the near future. Recently, the combined deep imaging of bone marrow and transgenic mouse models for lineage tracing identified a mechanosensitive perivascular stem cell niche (the surrounding microenvironment) at diaphysis—the midsection shaft of the long bone. Specifically, the Oln^+^ stromal cells were found exclusively in bone marrow peri‐arteriolar niche—selectively harboring around arterioles and arteries but not sinusoids.^[^ ^2^, ^7^ ^]^ At the molecular scale, the mechanosensitive ion channel Piezo1 was shown to play a critical role in maintaining Oln^+^ cell niche for intramedullary osteogenesis and lymphopoiesis.^[^ ^2^, ^8^ ^]^ This peri‐arteriolar niche was reduced during aging and can be expanded with increased mechanical loading such as running.^[^ ^2^ ^]^ Loss of mechanical loading, either with hindlimb unloading or Piezo1 channel deletion reduced Oln^+^ cell proliferation, bone mineral density, and lymphoid progenitor frequency.^[^ ^2^, ^8^ ^]^ Nevertheless, how forces induced by walking or running are transmitted from hard compact bone into the soft bone marrow still remain elusive around this fascinating mechanobiology. Significant challenges reside in directly interrogating and dissecting various mechanical effects of exercise on bone marrow in vivo.^[^ ^9^, ^10^, ^11^ ^]^

The transverse nutrient vessels, particularly arterioles that have stiffer and thicker vessel walls, are often present in the bone marrow by passing through the bone.^[^ ^12^ ^]^ During exercise, these vessels are effective at transmitting vibration‐induced mechanical stimuli from the bone cortex into the bone marrow,^[^ ^7^, ^13^, ^14^, ^15^ ^]^ thereafter stimulating the peri‐vascular niche and determining the stem cell fates.^[^ ^2^ ^]^ Additionally, A widely discussed hypothesis indicates that the intravascular pulse pressure (PP, defined as the difference between systolic and diastolic blood pressures) increases during exercise. It may also expand the blood vessels inside bone marrow, propagating the contraction force from the heart to the perivascular stromal cells.^[^ ^16^, ^17^ ^]^ Another theory suggests that bone may deform during exercise, therefore propagating the external mechanical stimuli to bend the vessels in the soft marrow.^[^ ^18^, ^19^ ^]^ Nevertheless, none of these hypotheses can be easily tested experimentally in vivo yet.

In the past decade, the computational biomechanics field has rapidly evolved due to significant advancement of digital imaging techniques, which allows realistic modeling of the anatomical features and mechanical behaviors of bone tissues under body exercises and bone diseases.^[^ ^20^, ^21^, ^22^, ^23^ ^]^ While most of these studies focus on the cortical bones, the recent breakthrough of tissue clearing techniques revolutionized the in vivo deep bone imaging technique, thereafter achieving unprecedented resolution of vasculature inside the bone marrow.^[^ ^2^, ^7^ ^]^ For computational biomechanics analysis, reconstructing characteristic vascular anatomy is a prerequisite. The early approaches conduct vascular reconstruction through 2D segmentation of magnetic resonance imaging (MRI)^[^ ^24^ ^]^ and micro‐computed tomography (CT) images,^[^ ^25^ ^]^ which are labor‐intensive, time‐consuming,^[^ ^26^ ^]^ and inevitably lose some important anatomical information.^[^ ^24^, ^25^ ^]^ The latest 3D reconstruction has incorporated more genuine geometric details thanks to the advancement of adjustable volumetric 3D techniques across different length scales.^[^ ^23^, ^27^, ^28^, ^29^, ^30^, ^31^ ^]^ Further, the elasticity of a blood vessel is known to be variable, which changes with its deformation due to the presence of its smooth muscle cells (hyperelastic). To describe the sophisticated elastic behavior of blood vessels, different constitutive models have been developed, such as i) Ogden^[^ ^32^ ^]^ that assumes linear strain energy density; ii) Mooney–Rivlin (3‐ and 5‐parameter)^[^ ^32^, ^33^ ^]^ derived from Ogden but more specific for small deformation scenarios, such as physiological blood vessel modeling without rapture; iii) Yeoh^[^ ^34^ ^]^ that simplifies the Mooney–Rivlin model from 3D to 2D; Other simplified models that assume uniaxial elasticity such as Saint–Venant–Kirchhoff^[^ ^35^ ^]^ and Neo–Hookean.^[^ ^36^ ^]^ For large blood vessels such as the aorta and carotid, the tissues are comprised of layers with different material properties, known to be heterogeneous.^[^ ^14^, ^15^, ^37^ ^]^ At smaller scales, the elasticity of arterioles and arteries in bone marrow (diameter < 50 µm) are more homogeneous.^[^ ^15^, ^38^ ^]^

Hereby, for the first time, we devised a one‐stop computational biomechanics framework on bone marrow vasculature. By taking advantage of the latest deep confocal images of the cleared tibia, we reconstructed the 3D vascular geometries in the presence of vessel wall thickness and intravascular pulse pressure. Treating the blood vessel walls as homogeneous,^[^ ^14^, ^37^, ^39^, ^40^ ^]^ we first created smooth 3D bone marrow vascular geometries and depicted the hyperelastic mechanical properties by implementing a 5‐parameter Mooney–Rivlin model. Then we performed finite element analysis (FEA) to thoroughly investigate the mechanical effects of exercise‐induced vibratory stretching on bone marrow vasculature and then quantitatively evaluate the mechanopropagation capability for arterioles, arteries, and sinusoids. Last but not least, using the same model, we examined the mechanical property changes of bone marrow vessels due to intravascular pulse pressure.

## 2 Results

### 2.1 3D Vascular Reconstruction and Finite Element Meshing from the Deep Bone Marrow Confocal Imaging

**Figure 1:**
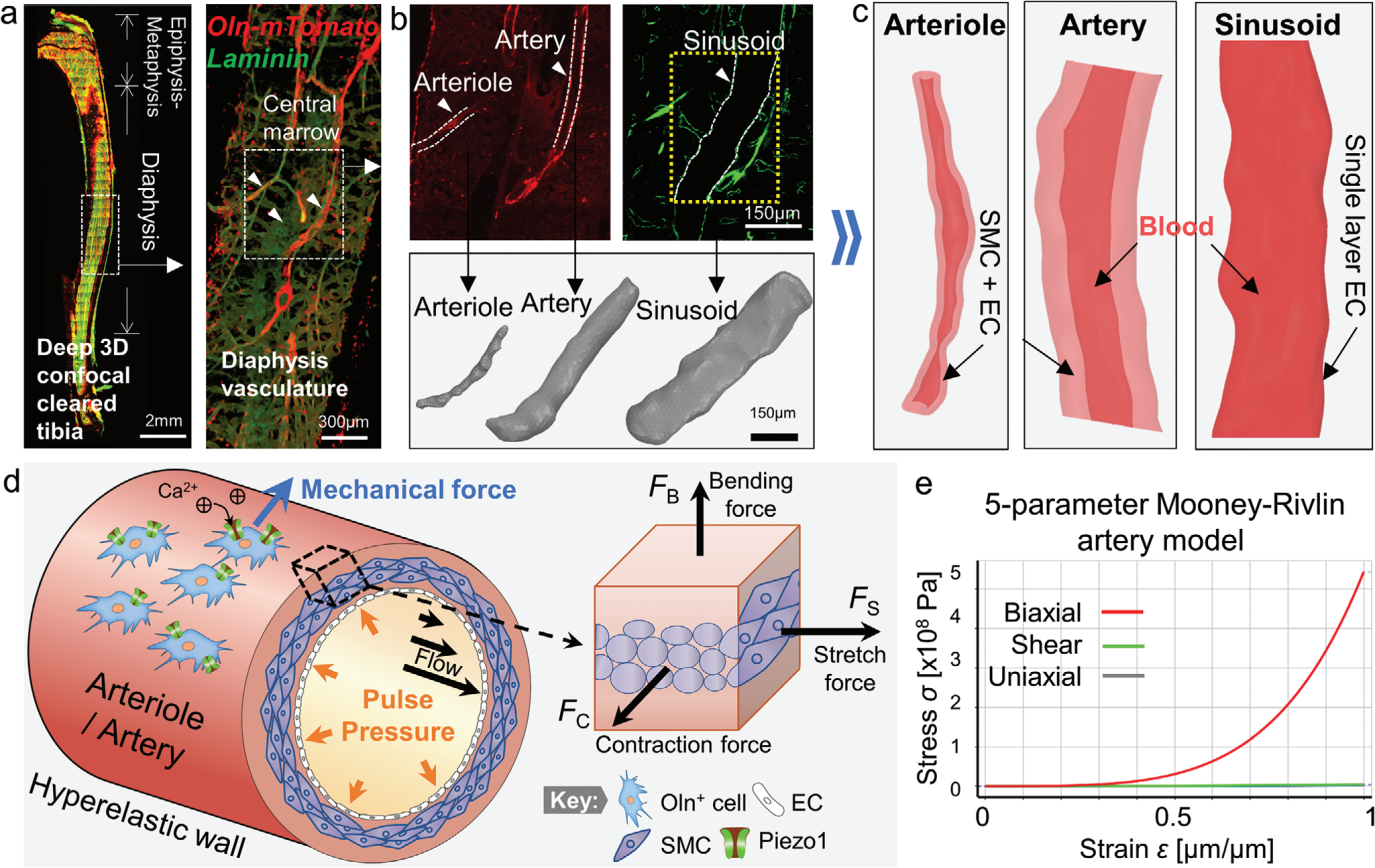
3D vascular reconstruction and biomechanical analysis configuration from the deep bone marrow confocal imaging. a) Deep 3D confocal scanned mouse tibia (left) and representative 3D rendered diaphysis bone marrow (right) showing *Oln^mT^* ^+^ cells (red) and basal lamina (green).^[^ ^2^ ^]^ b) Peri‐arteriolar *Oln^mT^* ^+^ cells (left, arrowhead) were stained to show the anatomy of arterioles and arteries. The basal lamina (right, yellow dot line) and the sinusoid lumens (white dash line) show the sinusoidal anatomy. The standard triangle language of the representative arteriole, artery, and sinusoid were extracted from the raw confocal images using Bitplane Imaris (bottom). Scale bar = 150 µm. c) The anatomical smooth reconstructed 3D structure of an arteriole, an artery, and a sinusoid. Note that the facades were converted to curvature‐based continuous surfaces in the presence of vessel walls using ANSYS SpaceClaim. The arteriolar and arterial wall consist of smooth muscle cells (SMC) and endothelial cells (ECs), while the sinusoidal wall consists only of EC without SMC. d) Schematics of force analysis of peri‐ and intra‐arteriolar/arterial structures. Note that the SMC (purple) in the middle of vessel walls contributes to vessel elasticity, while the innermost layer in contact with blood comprises a lining layer of EC. The elasticity of small arterioles and arteries in bone marrow (diameter < 50 µm) are assumed to be homogeneous. The forces/stress acting on the arteriolar/arterial wall are induced by intravascular pulse pressure, cortical bone bending, and vibratory stretch force. The Oln^+^ cells on the outer surface of the arteriole/artery sense the mechanical forces (blue arrow) via the Piezo1 ion channel. e) The stress and strain plot of the 5‐parameter Mooney–Rivlin artery model, in which the elasticity is variable with respect to the strain *ε* (Equation (1)). Note that the shear (green) and uniaxial (gray) curves were superimposed.

Due to the opaqueness of densely packed cells, it is technically challenging over decades to visualize the anatomical vasculature and rare stem cell niches in the deep bone marrow. To this end, the recent advance in optical clearing techniques, specifically the modified Murray's clear (1:2 Benzyl Alcohol: Benzyl Benzoate; BABB), has enabled deep imaging of bone marrow vasculatures inside the mouse tibia and femur with unprecedented depth (>200 µm) and resolution (submicron) (**Figure** **1**a, left).^[^ ^2^, ^7^ ^]^ When combined with the knock‐in mice *Oln*‐*mTomato* (*Oln^mT^*), the deep bone imaging experiments localized the key mechanosensitive niche *Oln^mT^* ^+^ cells, mainly peri‐arteriolar and peri‐arterial in bone marrow (Figure 1a,b, red) but not associated with the laminin‐stained sinusoids (Figure 1a,b, green) and other vessel types.^[^ ^2^ ^]^ Notably, such immunofluorescence imaging also revealed the detailed anatomy of the bone marrow vasculature, where the arteries and sinusoids traverse longitudinally along the bone shaft, while the transverse arterioles, often serving as nutrient vessels, pass through cortical bone to enter the bone marrow (Figure 1a).^[^ ^2^ ^]^ Although being sufficient to determine functional phenotypes for biologists, the intrinsic limitations, with respect to the intensity heterogeneity and fluorescence discontinuity in these 3D sectioned confocal images (Figure 1b, bottom), prevent precise digital vascular reconstruction and the subsequent computational modeling across the compact bone (hard tissue) into the deep bone marrow (soft tissue).

To address this issue, we devised a one‐stop computational method using the hyperelastic model to smoothly reconstruct high‐precision 3D anatomies of bone marrow vessels (Figure 1b,c). In the first step, the marrow vessel geometries were extracted from the raw confocal images of the cleared tibia diaphysis (Figure 1b). For digital reconstruction on arterioles and arteries, which are well demarcated by the *Oln^mT^* fluorescence reporter, we directly converted the imaged confocal points into the Standard Triangle Language (STL) format using Bitplane Imaris (Figure 1b, bottom). Notably, due to the limitation of deep bone immunostaining, the confocal images of sinusoids stained by laminin or endomucin^[^ ^2^ ^]^ (Figure 1b, green) usually lack clear and continuous luminal boundary. Thus, we instead reconstructed the surrounding contours around the sinusoids (Figure 1b, yellow dotted lines), followed by Boolean subtraction to inversely rebuild the sinusoids lumens (Figure 1b, broken white lines). In the second step, we converted the STL into curvature‐based continuous surfaces (see Section 4) (Figure 1c). To reconstruct the volumetric geometries, we further considered the vessel wall thickness and blood contents, then incorporated corresponding morphological parameters and mechanical properties (**Table** **1**; Figure S1a, Supporting Information).^[^ ^41^, ^42^, ^43^, ^44^, ^45^, ^46^ ^]^

**Table 1:** In vivo physical parameters for different bone tissue components. Columns from left to right are: Tissue components used in the 3D reconstruction and finite element analysis; Thickness range; Model elasticity assumptions; Young's modulus; Corresponding references. In all simulations, the arteriolar and arterial walls were set as constant thicknesses (see Experimental Section). All hyperelastic vessels were modeled using the 5‐parameter Mooney‐Rivlin model, which is more suitable for physiological conditions. Young's modulus of linear elastic blood was set as a small number ≈0 in all simulations. The endothelium was ignored in the simulation. Note that due to the scale of mice bone marrow vessels, it is impossible to measure their elasticity experimentally. Therefore we used the elastic moduli for humans to model the arteriole and artery walls (see Experimental Section)

Tissue components	Thickness [µm]	Elasticity	Young's modulus	References
Arteriole wall	5–15	Hyperelastic	20–200 MPa	[41, 42, 43]
Artery wall	15–20	Hyperelastic	20–200 MPa	[41, 42, 43]
Blood (fluid)	7.5–60	Mass only	≈0 kPa	[41, 44]
Sinus wall	≈0	Ignored	≈0 kPa	[41, 44, 45]
Bone Marrow	3000	Linear elastic	10 kPa	[41, 44, 46]

To prepare the biomechanical finite elements simulation, the vascular geometries were then meshed with ultrafine grids utilizing tetrahedron elements^[^ ^47^, ^48^ ^]^ (Figure S1b, Supporting Information). Due to the presence of smooth muscle cells, the thick‐walled arteries and arterioles are known to have complex hyperelastic mechanics where the stiffness increases with deformation (Figure 1d).^[^ ^14^, ^15^, ^41^ ^]^ In contrast, the thin‐walled sinusoids consist of discontinuous endothelium without smooth muscle cells.^[^ ^14^, ^15^, ^41^ ^]^ To recapitulate the hyperelastic mechanical properties (Table 1), the 5‐parameter Mooney–Rivlin model was implemented to simulate the mechanical responses, distribution, and propagation over time (Figure 1e)^[^ ^43^ ^]^

(1) $$\sigma =\frac{dW}{d\varepsilon }$$

where *σ* is the stress components (with respect to normal stress and shear stress), *W* is the strain energy density (see Section 4) and *ε* is the strain. In contrast, the bone marrow was characterized using a 3D linear elastic model (Table 1)^[^ ^14^ ^]^

(2) $$\begin{array}{c} \sigma_{1}-\nu \;\left(\sigma_{2}+\sigma_{3}\right)\;=\;E\times \varepsilon_{1}\\ \sigma_{2}-\nu \;\left(\sigma_{1}+\sigma_{3}\right)\;=\;E\times \varepsilon_{2}\\ \sigma_{3}-\nu \;\left(\sigma_{1}+\sigma_{2}\right)\;=\;E\times \varepsilon_{3} \end{array}$$

where *E* is Young's modulus and *ν* is Poisson's ratio. To take into account of both normal and shear stresses, we used von Misses stress to characterize blood vessel mechanopropagation in the rest of the study.

### 2.2 Transverse Vessels Mediated Vibratory Stretching Mechanopropagation

**Figure 2:**
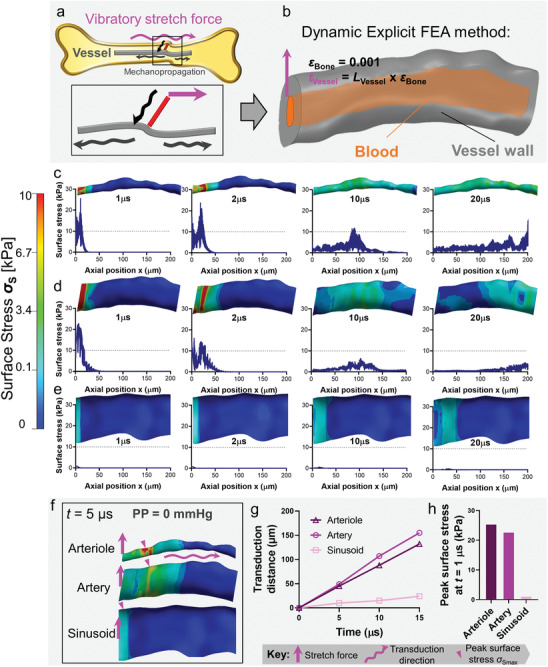
3D simulation of transverse vessels mediated vibratory stretching mechanopropagation. a) Schematic of exercise‐induced vibratory stretch force at the interface between the cortical bone and a transverse vessel. We used the dynamic explicit FEA method to simulate the time‐dependent moving process. b) Boundary conditions imposed by a pulsatile stretch at the bone‐vessel interface. Not the intravascular blood was also modeled in this circumstance (orange).^[^ ^51^ ^]^ c–e) The contour map of surface von Misses stress *σ*_S_ in transverse arteriole (c), bone marrow artery (d), and sinusoid (e) at *t* = 1, 2, 10, 20 µs (the first, second, third, and fourth column from left to right) after imposing a mechanical pulsatile stretch without pulse pressure. Note that the color bar is non‐linearly distributed in order to display small stress areas for sinusoids (*σ*_S_ ≤ 0.1 kPa). The artery displays the fastest while the sinusoid displays the slowest in mechanopropagation. The impacted area (red and green) and zero state area (blue) indicate that the artery and arterial are more efficient in propagating forces than the sinusoid. f) The contour of surface Von Misses stress *σ*_S_ in the transverse arteriole, the bone marrow artery, and sinusoid at *t* = 5 µs after imposing a mechanical pulsatile stretch without pulse pressure. g) Propagation distance of the pulsatile stretch indicated by *σ*_Smax_ over time in a transverse arteriole, the target bone marrow artery, and sinusoid. h) Peak surface Von Misses stress *σ*_Smax_ of arteriole, artery, and sinusoid at *t* = 1 µs.

During body exercise, substantial vibration frequently stretches the bone endosteum^[^ ^49^ ^]^ (**Figure** **2**a), leading to movement‐induced mechanical stimuli that can be transmitted into the bone marrow rather than being absorbed entirely by cortical bone. Several shreds of evidence support the role played by the transverse nutrient vessels (Figure 2a, red) in such mechanopropagation.^[^ ^11^, ^50^ ^]^ The complex cyclic vibratory stretches can be decoupled into single tensile pulses.

To this end, we first applied a tensile stretch pulse to one side of a transverse nutrient vessel (arteriole), as well as to an artery and a sinusoid (Figure 2a,b, magenta arrow). The rest of the vessel structures remain unconstrained. It is worth noting that the mechanical property of arterioles and arteries are determined by their vessel walls, while that of the sinusoid is determined by the surrounding marrow (Table 1). All the vessels were reconstructed with a constant wall thickness (cf. Figure 1c, pale red) and intravascular mechanical properties matching the blood content (cf. Figure 1c, dark red). The dynamic explicit FEA (Figure 2b) was employed here to simulate this moving process with 100 000 time step.

To characterize the dynamic process of vascular mechanopropagation, we denoted the magnitude of peak surface Von Misses stress as *σ*_Smax_, and the propagation velocity (Figure 2f, magenta arrowhead) as *v*_S_, respectively. Our FEA simulation indicated that as *σ*_Smax_ is propagating along the longitudinal direction for all three types of vessels (Figure 2c–e; Video S1, Supporting Information). The artery exhibited the fastest mechanopropagation, which only took *t* = 18 µs for *σ*_Smax_ to travel the entire 200‐µm vessel segment. During the same period, the *σ*_Smax_ traveled 185 µm on an arteriole, whereas only 45 µm on a sinusoid as the slowest (Figure 2c–e,g). Interestingly, the average speeds of mechanopropagation were *v*_S_ = 8.8 and 10.3 µm µs^−1^ on an arteriole and artery, respectively, equivalently 4.5‐ and 5.4‐folds relative to *v*_S_ = 1.6 µm µs^−1^ on a sinusoid (Figure 2g). Moreover, the *σ*_Smax_ of arteriole and artery were 24‐ and 20‐fold higher than that of the sinusoid at *t* = 20 µs, respectively. Notably, the thinner‐walled arteriole exhibited a higher *σ*_Smax_ compared to the thicker‐walled artery (Figure 2h, 25.5 vs 22.8 kPa). The *σ*_Smax_ also decreased over time, reducing from *σ*_Smax_ = 25.5 kPa at *t* = 1 µs to 10.54 kPa at *t* = 20 µs for arteriole, and from 22.8 kPa at *t* = 1 µs to 4.32 kPa at *t* = 20 µs for artery (Figure 2c–e). In conclusion, these results elucidate that the arterioles and arteries are more capable of propagating mechanical forces into deep bone marrow than sinusoids. The magnitude of peak stress is also affected by the vessel wall thickness.

### 2.3 Intravascular Pulse Pressure Induced Mechanopropagation

**Figure 3:**
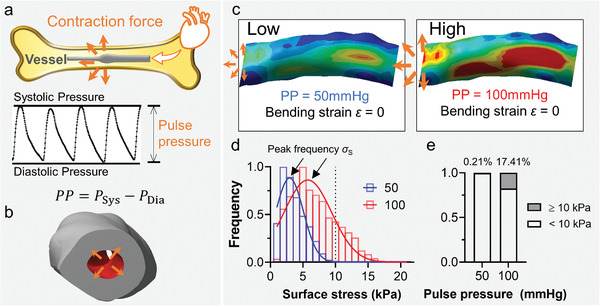
3D simulation of intravascular pulse pressure‐induced contracting mechanopropagation. a) Schematic of intravascular pulse pressure (PP) induced endovascular contraction force (top), where the pulse pressure is the difference between systolic (*P*_Sys_) and diastolic (*P*_Dia_) pressure (bottom). b) Defining the pulse pressure that models the endovascular surfaces under walking and running states. Note that the pulse pressure is imposed perpendicularly toward endovascular surfaces. c) The contour of surface Von Misses stress *σ*_S_ in the artery under 50 (left) and 100 (right) mmHg pulse pressure alone. d) *σ*_S_ distribution and Gaussian curve fitted histogram under PP = 50 (blue) and 100 (red) mmHg. e) Fraction of surface area where contractile stress is higher and lower than 10 kPa under 50 and 100 mmHg pulse pressure without stretch.

After the digital vascular reconstruction and vibratory simulation, we next tested a hypothesis that the intravascular pulse pressure‐induced contraction force regulates the vascular mechanopropagation. Studies suggest that the pulse pressure increases by nearly twofold while experiencing heavy exercises, from 40 to 100 mmHg (**Figure** **3**a).^[^ ^52^, ^53^ ^]^ Such increment significantly expands the blood vessels, therefore propagating the contraction forces into the bone marrow. Experimentally, the tibia and femur are longitudinally cut in half, dehydrated, optically cleared, then stained^[^ ^2^ ^]^ before the deep bone marrow imaging. Such processing inevitably reduces the sizes of vasculature^[^ ^2^, ^7^ ^]^ and distorts the vessels’ physiological geometries due to the loss of intravascular pulse pressure.^[^ ^2^ ^]^ In addition, the pulse pressure also results in volumetric expansion for arteries, therefore increasing their Young's modulus (hyperelasticity).

Hereby, we computationally introduced intravascular pulse pressure on a target artery inside the deep bone marrow (Figure 3a,b, gray) to restore the physiological geometries and mechanical properties (Equation (3)). Pulse pressures at 50 and 100 mmHg were defined on the endovascular surfaces to model the mild (walking) and strenuous (running) exercising states, respectively (Figure 3b).

The pressure was numerically applied to the endothelium of a 3D reconstructed target artery. The distribution of surface Von Misses stress *σ*_S_ was sampled on the outer surface of this target artery using a 10 µm × 10 µm sampling grid (Figure 3; Figure S1d, Supporting Information). The contour map of *σ*_S_ was then obtained under the mild (50 mmHg) and strenuous (100 mmHg) exercise pulse pressures (Figure 3c). Interestingly, *σ*_S_ increased proportionally with the pulse pressure (Figure 3c,d), ranging from 0 to 12 kPa at 50 mmHg and from 0 to 22 kPa at 100 mmHg (Figure 3d). The most frequent *σ*_S_ increased by twofold from 3 to 6 kPa (Figure 3d, arrow). In other words, the higher the pulse pressure, the larger the possibilities (Figure 3e; 17.41% vs 0.21%) of experiencing high contractile stress (*σ*_S_ ≥ 10 kPa). Thus, this FEA result demonstrates that strenuous exercise promotes vascular mechanopropagation by elevating intravascular pulse pressure.

### 2.4 Cortical Bone Bending Mediated Perivascular Mechanopropagation

**Figure 4:**
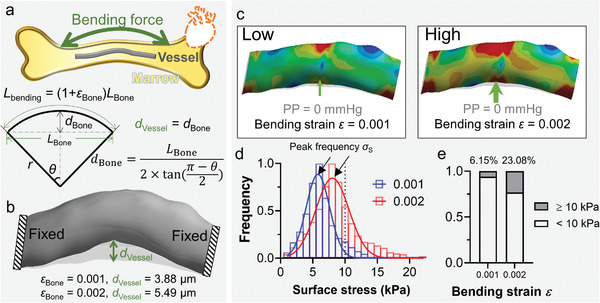
3D simulation of cortical bone propagated perivascular bending mechanopropagation. a) Schematic of exercise‐induced bending force in bone and its propagation to bone marrow vessels. It is assumed that the vascular deformation *d*_Vessel_ is equal to the bone deformation *d*_Bone_. The vessel deformation is positively correlated (see Section 4) to the macro deformation *L*_bending_ and inversely proportional to the microstrain *ε*_Bone_. b) The two ends of the reconstructed vessel were fixed before finite elements simulation, with a displacement of *d*_Vessel_ = 3.88 or 5.49 µm in the middle of the vessel. c) The contour map of *σ*_S_ of the artery under *ε* = 0.001 (left) and 0.002 (right) bending strain without pulse pressure. d) *σ*_S_ distribution and Gaussian curve fitted histogram under *ε* = 0.001 (blue) and 0.002 (red) bending strain without pulse pressure. e) Fraction of surface area where bending stress is higher and lower than 10 kPa under *ε* = 0.001 and 0.002 bending strain without pulse pressure.

The exercise‐induced bone deformation plays a vital role in propagating bending force from the cortical bone.^[^ ^54^ ^]^ Another well‐known hypothesis of bone marrow vasculature mechanopropagation is that the adapted marrow would bend with the cortical bone thus bend the blood vessels inside (**Figure** **4**a, gray).^[^ ^18^, ^19^, ^54^ ^]^ Interestingly, such bending was reported to correlate with the bone mass increase in vivo.^[^ ^18^, ^54^ ^]^ To model the overall bending deformation, denoting a displacement in the middle (*d*_Vessel_), we defined an extreme condition of vessel bending effect by fixing the two ends of a target artery before finite elements simulation (Figure 4a,b).

To test this hypothesis and examine the cortical bone bending mediated mechanopropagation on a target artery, we introduced the bending strain at *ε* = 0.001 and 0.002 to model vascular deformation (Figure 4b) .^[^ ^18^, ^19^, ^54^ ^]^ Surface bending stress *σ*_S_ derived from the two bending strains *ε* = 0.001 and 0.002 were measured at the outer surface of the vessel with both longitudinal ends fixed (Figure 4c). Both *σ*_S_ distributed in single population fit by the Gaussian, ranging from 0 to 10 kPa for *ε* = 0.001 (Figure 4d, blue) and from 0 to 24 kPa for *ε* = 0.002 (Figure 4d, red). The most frequent *σ*_S_ peaked at 6 and 8 kPa for *ε* = 0.001 and 0.002, respectively (Figure 4d, black arrow). In other words, the higher the bending strain, the larger the possibility of experiencing high bending stress *σ*_S_ ≥ 10 kPa (Figure 4e; 23.08% vs 6.15%). To this end, this FEA result demonstrates that the bending mediated perivascular mechanopropagation is increased when the cortical bone is deformed.

### 2.5 Coupling Intravascular Pulse Pressure with Bending and Stretching Mechanopropagation

**Figure 5:**
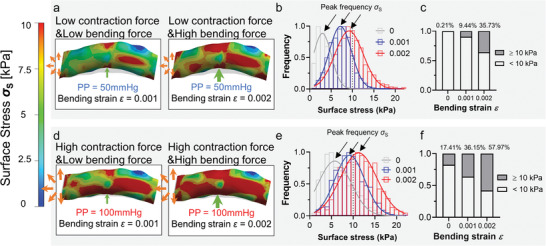
The synergistic effect of intravascular pulse pressure and perivascular bending on mechanopropagation. a) The contour map of surface Von Misses stress *σ*_S_ of the artery under *ε* = 0.001 (left) and 0.002 (right) bending strain and pulse pressure of 50 mmHg. b) *σ*_S_ distribution and Gaussian curve fitted histogram under *ε* = 0 (gray), 0.001 (blue), and 0.002 (red) bending strain and pulse pressure of 50 mmHg. c) Fraction of surface area where the surface Von Misses stress *σ*_S_ are higher and lower than 10 kPa under *ε* = 0, 0.001 and 0.002 bending strain and pulse pressure of 50 mmHg. d) The contour map of *σ*_S_ of the artery under *ε* = 0.001 (left) and 0.002 (right) bending strain and pulse pressure of 100 mmHg. e) *σ*_S_ distribution and Gaussian curve fitted histogram under *ε* = 0 (gray), 0.001 (blue) and 0.002 (red) bending strain and pulse pressure of 100 mmHg. c) Fraction of surface area where the surface Von Misses stress *σ*_S_ are higher and lower than 10 kPa under *ε* = 0, 0.001 and 0.002 bending strain and pulse pressure of 100 mmHg.

The intravascular pulse pressure presents during the lifetime of human and mice. It is important to consider such effects in all vascular biomechanics modeling. To this end, we examined the synergistic effect of intravascular pulse pressure coupled with cortical bending on the target artery (**Figure** **5**). When PP = 50 mmHg (Figure 5a), the surface Von Misses stress *σ*_S_ derived from bending strain *ε* = 0.001 and 0.002 distributed in a single population. An increasing trend of *σ*_S_ was observed to peak at 16 and 22 kPa for *ε* = 0.001 (Figure 5b, blue) and *ε* = 0.002 (Figure 5b, red), respectively. The possibility of high bending stress (*σ*_S_ ≥ 10 kPa) increased by nearly threefold (Figure 5c, 35.73% vs 9.44%). When PP = 100 mmHg (Figure 5d), a similar increasing trend was observed (Figure 5e). The possibility of high bending stress increases from 1/3 to 1/2 (Figure 5f, 36.55% vs 57.57%). Notably, the effect of bending dominated over that of intravascular pulse pressure on surface Von Misses stress distribution when the bending strain increases to a strenuous level (*ε* = 0.002). These findings correlate with the well‐established bone loading‐mass experiments,^[^ ^8^, ^18^, ^54^, ^55^ ^]^ suggesting that only stretching over a certain threshold (*ε* = 0.001) could stimulate the increment of bone mass.

**Figure 6:**
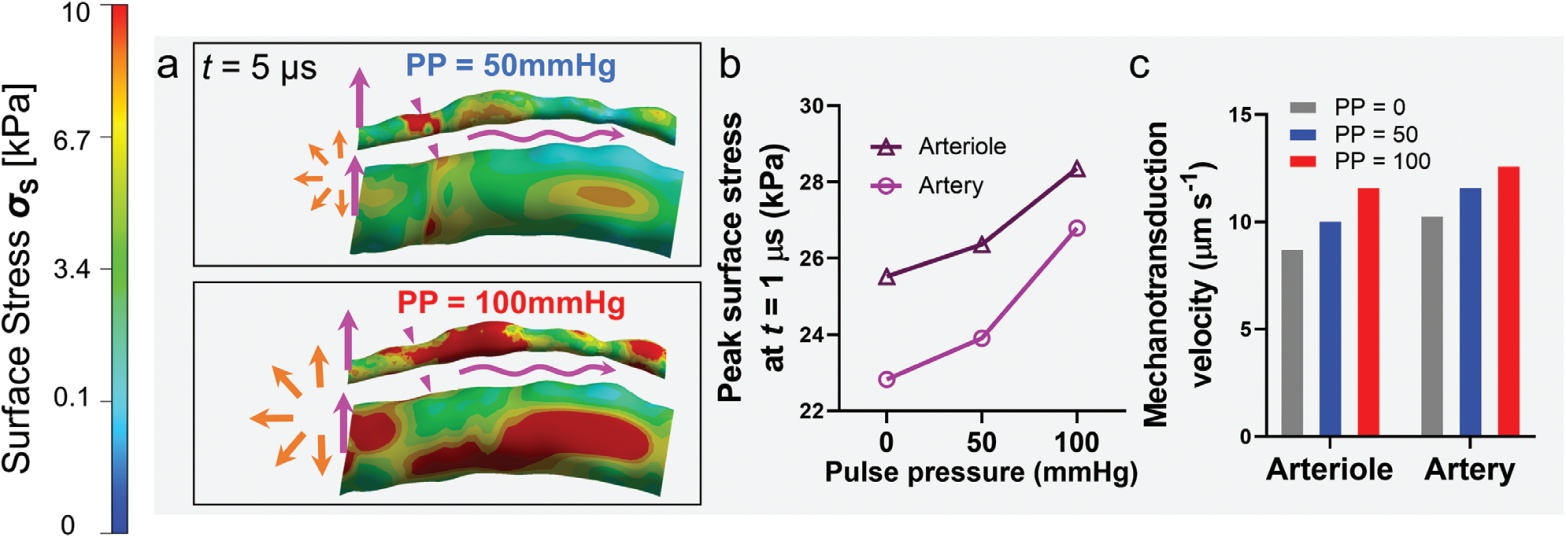
3D simulation of the mechanopropagation of transverse vessels mediated vibratory stretching coupled with intravascular pulse pressure. a) The contour of *σ*_S_ in the arteriole and artery at *t* = 5 µs after imposing a single mechanical pulsatile stretch under pulse pressures of 50 (top) and 100 mmHg (bottom). b) *σ*_Smax_ of the arteriole, artery, and sinusoid at *t* = 1 µs under pulse pressures of 0, 50, and 100 mmHg. c) The average mechanopropagation velocity *v*_S_ of the arteriole and artery under pulse pressures of 0, 50, and 100 mmHg.

Furthermore, we investigated the vibratory stretching effect coupled with the mild and strenuous exercise pulse pressures (**Figure** **6**a). The overall *σ*_Smax_ increased with pulse pressure (Figure 6b; Figure S2, Supporting Information). Notably, when the pulse pressure (PP = 50 and 100 mmHg) was considered in the vibratory FEA simulation, the *σ*_Smax_ at *t* = 1 µs increased by 3% and 11% for arteriole, and 5% and 17% for artery, respectively (Figure 6b). The diminishing *σ*_Smax_ was also observed in the presence of intravascular pulse pressure. At PP = 50 mmHg, the *σ*_Smax_ of arteriole and artery decreased from 26.4 and 23.9 kPa at *t* = 1 µs to the baseline at *t* = 20 µs, respectively (Figure S2a,b, Supporting Information). A similar decreasing trend was observed at PP = 100 mmHg (Figure S2c,d, Supporting Information).

Last but not least, we examined the pulse pressure effects on mechanopropagation velocity. When pulse pressure increased from 0 to 50 and 100 mmHg, the average velocities exhibited 15% (*v*_S_ = 8.8 to 10.1 µm µs^−1^) and 33% (*v*_S_ = 8.8 to 11.7 µm µs^−1^) faster in an arteriole (Figure 6c). Similar results and trends were observed with an artery. As the pulse pressure remains nearly zero for sinusoids under all physiological conditions,^[^ ^13^, ^14^ ^]^ the synergistic effect can be neglected.

Taken together, our computational studies support that stiffer‐walled vessels, including arterioles and arteries, are better at transmitting mechanical stimuli than softer‐walled vessels.^[^ ^41^ ^]^ Sinusoidal blood vessels, which represent a majority of blood vessels in the bone marrow, have thin walls with discontinuous endothelium and no smooth muscle.^[^ ^14^, ^15^, ^41^ ^]^ This may explain why mechanosensitive *Oln^mT^* ^+^ niches localize around arterioles and arteries (Figure 1a). Although vibratory stretching plays a significant role, the exercise‐induced pulse pressure synergistically facilitates vascular mechanopropagation in an incremental manner.

## 3 Discussion

Although the blood vessels (Figure 1a, arrowhead; Figure 1b, white dash line) inside deep bone marrow are known to be critical for the perivascular mechanosensing,^[^ ^14^, ^44^, ^49^, ^56^ ^]^ how the mechanosensitive perivascular niches are uniquely maintained by exercise and mechanical loading is a fundamental open question in the field. To our knowledge, techniques have not yet been developed to measure the surface stress of bone marrow blood vessels in vivo without disrupting tissue architecture. As a result, most numerical modeling works on skeletal biomechanics were conducted at cortical bone.^[^ ^18^, ^19^, ^54^ ^]^ To this end, our computational study presents novelties in: 1) Reconstruct the in vivo blood vessels on micron scales by overcoming the fluorescence discontinuity in confocal images of deep bone marrow; 2) Restore the physiological bone marrow vascular geometries and mechanical properties by introducing intravascular pulse pressure; 3) Simulate three hypothetical exercise‐induced mechanopropagation mechanisms across bone marrow arterioles, arteries, and sinusoids. It must be pointed out that the applicability of our computational biomechanics framework is not limited to confocal images but has a broader potential in analyzing 3D vascular and tissue anatomies obtained from other medical imaging modalities such as CT^[^ ^57^ ^]^ and MRI.^[^ ^58^ ^]^ Our in silico approaches will not only accelerate the shift from idealized/generic to subject/patient‐specific modeling but also provide large‐scale phenotypic data in the context of bone marrow mechanobiology.

Experimental‐wise, although absent direct tools, the external bone loading methods represent the field standard to investigate how exercise affects bone mass and mineral density.^[^ ^18^, ^55^, ^59^ ^]^ From the literature, it has been observed that the peak bone surface strain (*ε*_max_) below 0.001 is associated with bone loss while *ε*_max_ above 0.001 leads to substantial periosteal and endosteal osteogenesis.^[^ ^18^, ^54^ ^]^ It also suggested that time scale and cyclic loads are critical for mechanopropagation, where time scales of the order of microsecond are too small for usual cell reaction time.^[^ ^2^, ^60^ ^]^ In this aspect, our numerical study demonstrates that the intravascular pulse pressure‐induced contraction alone has a limited effect in enhancing surface Von Misses stress *σ*_S_ on a target artery (<20%; Figure 3). Nonetheless, when coupled with external bending or vibratory stretching, the *σ*_S_ and mechanopropagation increased excessively (Figures 4 and 5). Such joint results suggest a synergistic but not dominant role of intravascular pulse pressure in skeletal mechanopropagation and osteogenesis.

The vibratory effect of movement‐induced mechanical loading would be expected to stretch arterioles at the endosteum. Since the arterioles are stiff‐walled but elastic, they are expected to transmit these mechanical stimuli into the marrow, stretching the membranes of peri‐arteriolar cells. It is also possible that arterioles passing through cortical bone are compressed by movement‐induced mechanical loading or by shear stresses outside of the bone, leading to local blood pressure changes that stretch the arterioles within the marrow. Thus, there are multiple, non‐exclusive possibilities on how movement‐induced mechanical changes inside or outside arterioles propagate into the marrow. It is also worth noting that our mechanopropagation analyses only tested against the blood vessels at the diaphysis, or compact bone (Figure 1a, upper segment),^[^ ^61^ ^]^ while the metaphysis consists of spongy structure, or cancellous bone tissues (Figure 1a, lower segment).^[^ ^2^ ^]^ To model more complex trabecular networks and the corresponding mechanobiology of stem cell niche at the metaphysis,^[^ ^61^ ^]^ additional assumptions and boundary conditions need to be considered in future works.

It is worth noting that exercises such as swimming are assumed to increase intravascular pulse pressure but with limited cortical bone bending or vibratory stretching effects. Interestingly, it is reported that swimming had more minor bone mineral density increments than treadmill running.^[^ ^59^, ^62^, ^63^ ^]^ Such observation correlates with our finding that the intravascular pulse pressure is not the determinate factor, but systematically contributes to regulating the mechanosensitive perivascular niches. At the same time, the elasticity and stiffness (e.g., Young's modulus) of vessels determine the magnitude and speed of stretch‐induced force propagation. Further investigation into the effects of geometric shape and location of the stenosis, corner, and bifurcation in blood vessels would potentially contribute to a more thorough understanding of vascular mechanopropagation. In the near future, the emerging microfluidic^[^ ^64^ ^]^ and bioprinting technologies^[^ ^65^ ^]^ could soon reconstruct sophisticated in vivo vasculatures on a chip,^[^ ^66^ ^]^ thereafter experimentally test the abovementioned vascular mechanopropagation hypotheses in vitro.

## 4 Experimental Section

### Governing Equations of Hyperelastic Modulus

To model the mechanical behavior of SMC‐rich arteriolar/arterial walls, a 5‐parameter Mooney–Rivlin constitutive model has been adopted here.^[^ ^67^ ^]^ The strain energy density *W* can be calculated as a scalar function of the right Cauchy–Green deformation tensor, *C*. The scalar function is composed of either the principal invariants or the principal stretches of deformation, both of which are derived from the right Cauchy–Green deformation tensor. The experimental data from the literature were used to calibrate an isotropic hyperelastic strain energy density function,^[^ ^43^ ^]^ where C10 = 0.115, C01 = −0.049, C20 = 1.403, C11 = −3.370, C02 = 2.201. The artery walls could fit a general polynomial isotropic constitutive equation as Equation (3).

(3) $$\begin{array}{ccc} W & = & \sum_{i+j=1}^{N}C_{ij}{\left(\bar{I_{1}}-3\right)}^{i}{\left(\bar{I_{2}}-3\right)}^{j}\\ & & +\sum_{i\;=\;1}^{N}\frac{1}{D_{i}}{\left(J-1\right)}^{2i}\vdots i,j\;=\;0,1,\text{…},N\vdots i+j\;=\;1,2,\text{…},N \end{array}$$

where *I*_1_ and *I*_2_ are defined as

(4) $$I_{1}=\lambda_{1}^{2}+\lambda_{2}^{2}+\lambda_{3}^{2}$$

(5) $$I_{2}=\lambda_{1}^{2}\times \lambda_{2}^{2}+\lambda_{1}^{2}\times \lambda_{3}^{2}+\lambda_{2}^{2}\times \lambda_{3}^{2}$$

here,$\;\lambda_{1}^{2}$, $\lambda_{2}^{2},\;$and $\lambda_{3}^{2}$ are the squares of the principal stretch ratios, linked by the relationship *λ*_1_ *λ*_2_ *λ*_3_ = 1 due to incompressibility.

### Smooth 3D Vascular Geometry Reconstruction

In order to extract the vascular geometries, confocal images of *Oln^mT^* ^+^ cells and laminin were obtained using LEICA SP8 (lsm format). The data was then rendered in 3D and analyzed using Bitplane Imaris v9.0.1 (at the workstation of Sydney Cytometry at Charles Perkins Centre, University of Sydney). The representative vessel structures were selected from the Imaris rendered file in this study (c.f. Figure 1a), where the target artery and sinusoid are located in the center of the medullary cavity and traverse longitudinally along the bone shaft, and the target nutrient arteriole is going through the compact bone from periosteum to marrow. The “Surface” function in Imaris was utilized for the identification of vessel structure and to convert confocal points into STL format. Specifically, a 3D “Region of Interest” with 200 × 150 × 20 µm was created around the target artery and nutrient arteriole with 1 µm rendering resolution (Figure 1b, bottom). For the target sinusoid, a 3D “Region of Interest” with 900 × 400 × 200 µm was created to reconstruct the surrounding basal lamina first, followed by a Boolean subtraction to obtain its inner lumen. The exported 3D structures with STL were then uploaded to SpaceClaim program (ANSYS Inc. 2020) and converted to curvature‐based continuous surfaces. To trim the sharp spikes and some osteocytes outliers, the auto‐fix tool was used within SpaceClaim. The manual adjustment was available to delete the sharp edges and branches caused by image noise (Figure 1b). A “shrink wrap” function was applied to create a uniform surface around the object, bridging covers on small holes with 4.2 µm gap size (the minimum size to be bridged). Then the whole structure was smoothed by adding 200% more triangular faces with 60° angle threshold. In order to create the vessel wall, a constant thickness shell was adopted by uniformly shrinking the vessel surface inward in line with the vessel diameter (Table 1). Finally, the auto‐skin function is used for both original and shrunk structures, followed by Boolean subtraction to generate the curvature‐based continuous 3D vessel anatomy in the presence of vessel walls (Figure S1a, Supporting Information).

### The Finite Element Analysis

After obtaining the curvature‐based continuous 3D vessel anatomy, the model was imported to ANSYS workbench for extensive mechanical analysis (ANSYS Inc. 2020). The 3D reconstructed artery, arteriole, and sinusoid lengths were 200, 200, and 300 µm, respectively. Meanwhile, the arterial, arteriolar, and sinusoidal wall thicknesses were 14.3, 5, and ≈0 (ignored) µm (Table 1).^[^ ^15^ ^]^ Different meshing algorithms from hexahedron to tetrahedron, and grid sizes from 1 to 10 μm  were tested for mesh independence analysis.^[^ ^68^, ^69^ ^]^ Uniformly distributed 3 µm sized tetrahedron (Figure S1b, Supporting Information) and 10 µm sized hexahedron elements (Figure S1d, Supporting Information) were adopted for dynamic and static FEA.

### Boundary and Loading Conditions

The boundary and loading conditions of the 3D reconstructed vessels are prescribed as the clamped fixture (the unmoved part), a vibratory pulse tensile force (Figure 2b, magenta arrow), intravascular pulse pressure (Figure 3b, red area) and the bending effect *d*_Vessel_ in the middle (Figure 4b, green arrow). The fixtures are located at both longitudinal sides of vessels for all three hypothetic case scenarios. The pulse pressure was applied perpendicular to the inner vascular surface. The bending effect was applied in the middle of the target artery. The transverse vibration pulse was used as 1 µm displacement in the first 1 µs at one longitudinal end of all three types of vessels.

The blood and vessels are assumed to contact in frictionless.^[^ ^51^ ^]^ For arterioles and arteries, the mechanical effect of marrow can be ignored compared to the stiffness of the arteriole (more than 100‐folds of marrow). For the sinusoids, the stiffness of their endothelial vessel wall can be ignored. The surrounding bone marrow was created as the boundary of the intervascular blood (Figure S1c, yellow, Supporting Information).

Generally, the bending strain on bone (*ε*_Bone_) can be assumed as per the change (Δ*L*_Bone_) in original length (*L*_Bone_) as,

(6) $$\varepsilon_{\mathrm{Bone}}=\frac{\Delta L_{\mathrm{Bone}}}{L_{\mathrm{Bone}}}$$

and the transverse displacement in the center can be calculated from the bending slope *θ* (in radian) as shown in Figure 4a,

(7) $$d_{\mathrm{Bone}}=\frac{L_{\mathrm{Bone}}}{2\;\times \tan\left(\frac{\pi -\theta }{2}\right)}$$

(8) $$\frac{r\times \theta }{r\times \sin\theta }=1+\varepsilon_{\mathrm{Bone}}$$

where *r* is the bending radius.

In this scenario, we simulate the maximum flexural stress generated by the bending process of the femur bone, which is the worst physiological case. It is assumed that the artery has the same bending curvature as bone. Thus, the transverse displacement can be given as *d*_Vessel_ = *d*_Bone_ (Figure 4b).

## Conflict of Interest

The authors declare no conflict of interest.

## Author Contributions

Y.C.Z. and L.A.J. designed the research plan. Y.C.Z. developed the reconstruction framework and performed the simulations. B.S. and R.S. provided the deep confocal images and biological insights. Y.Z., F.J., C.W., B.W., and Q.L. provided critical advice. Y.C.Z. and L.A.J. wrote the manuscript. All authors reviewed the final manuscript.

## Supporting information

Supporting Information

Supplemental Video 1

## Data Availability

The data that support the findings of this study are available from the corresponding author upon reasonable request.
