# Supplementary material for: A Novel Computational Biomechanics Framework to Model Vascular Mechanopropagation in Deep Bone Marrow
Source: Adv Healthc Mater. 2023 Jan 8;12(8):2201830. doi: 10.1002/adhm.202201830 (PMC11469229; doi:10.1002/adhm.202201830)
Supplement: Supplementary file 1 — Supporting Information [file ADHM-12-2201830-s002.pdf]

Title: A novel computational biomechanics framework to model vascular mechanopropagation in deep bone marrow

Yunduo C Zhao[1,2,3], Yingqi Zhang[1,2,3], Fengtao Jiang[1,2,3], Chi Wu[4], Boyang Wan[4], Ruhma Syeda[5], Qing Li[4], Bo Shen[6,7,8], Lining A Ju[1,2,3*]

[1] School of Biomedical Engineering, The University of Sydney, Darlington, NSW, 2008, Australia

[2] Charles Perkins Centre, The University of Sydney, Camperdown, NSW, 2006, Australia

[3] The University of Sydney Nano Institute (Sydney Nano), The University of Sydney, Camperdown, NSW 2006, Australia

[4] School of Aerospace, Mechanical and Mechatronic Engineering, The University of Sydney, Darlington, NSW, 2008, Australia

[5] Department of Neuroscience, University of Texas Southwestern Medical Center, Dallas, TX 75235, USA.

[6] Children's Research Institute and the Department of Pediatrics, University of Texas Southwestern Medical Center, Dallas, TX 75390, USA.

[7] National Institute of Biological Science, Zhongguancun Life Science Park, Beijing, 102206, China.

[8] Tsinghua Institute of Multidisciplinary Biomedical Research, Tsinghua University, Beijing, 102206, China.

* Correspondence: arnold.ju@sydney.edu.au

Supplementary Figures

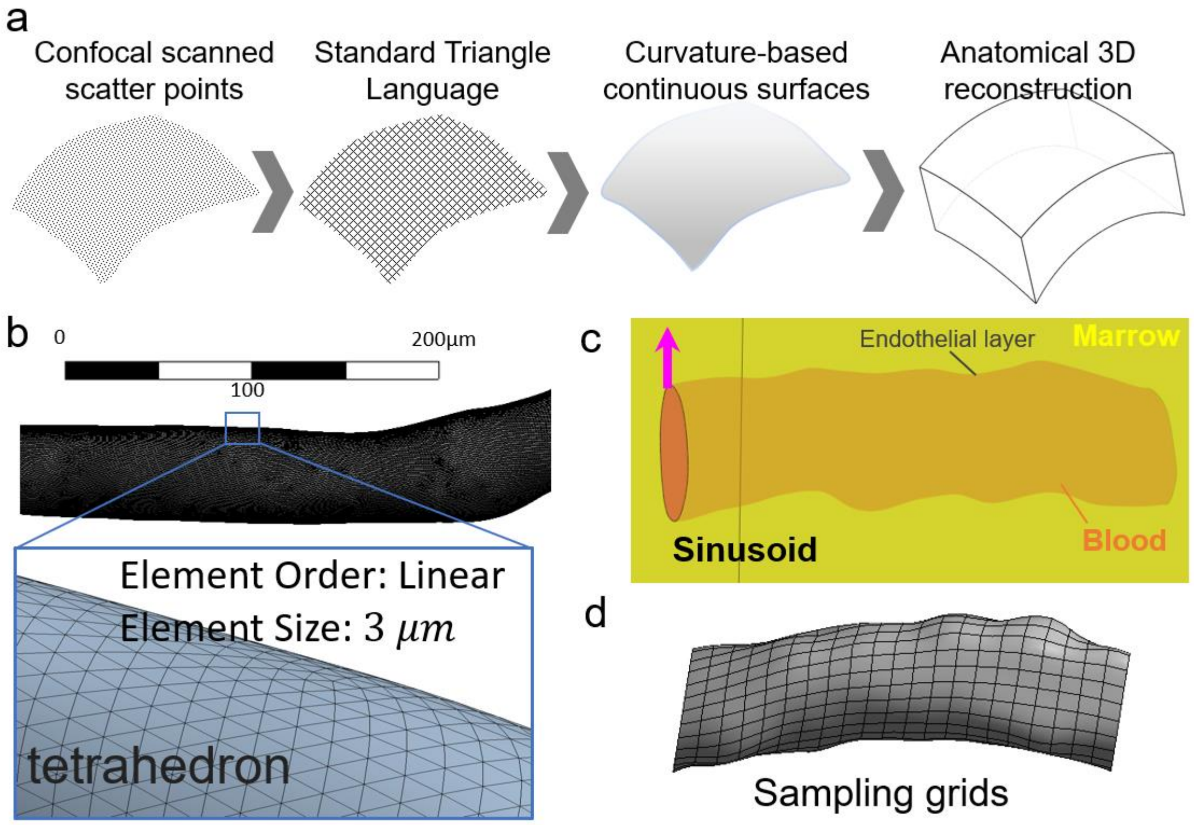

Figure S1. FEA data analysis. a) Smooth 3D reconstruction of the volumetric geometries of the bone marrow vessels. The confocal scanned scatter points were first reconstructed into Standard Triangle Language STL in Bitplane Imaris. Such facades were then converted into curvature-based continuous surfaces in ANSYS SpaceClaim. Finally, the vessel walls were reconstructed according to the corresponding morphological parameters from the literature (cf. Table. 1). **b)** ANSYS finite element meshing scheme for a representative 3D reconstructed artery. For finite element analysis, the entire reconstructed artery was meshed into 1,098,263 tetrahedron elements. **c)** Schematic showing the single endothelial-layered sinusoid, intravascular blood (*orange*), and the surrounding bone marrow (*yellow*). Note that the vessel wall thickness for sinusoids was set to 0 in the present simulation. **d)** A 10 μm × 10 μm sampling grid on the outer surface of the target artery for modeling the surface stress σ_S distribution and frequency.

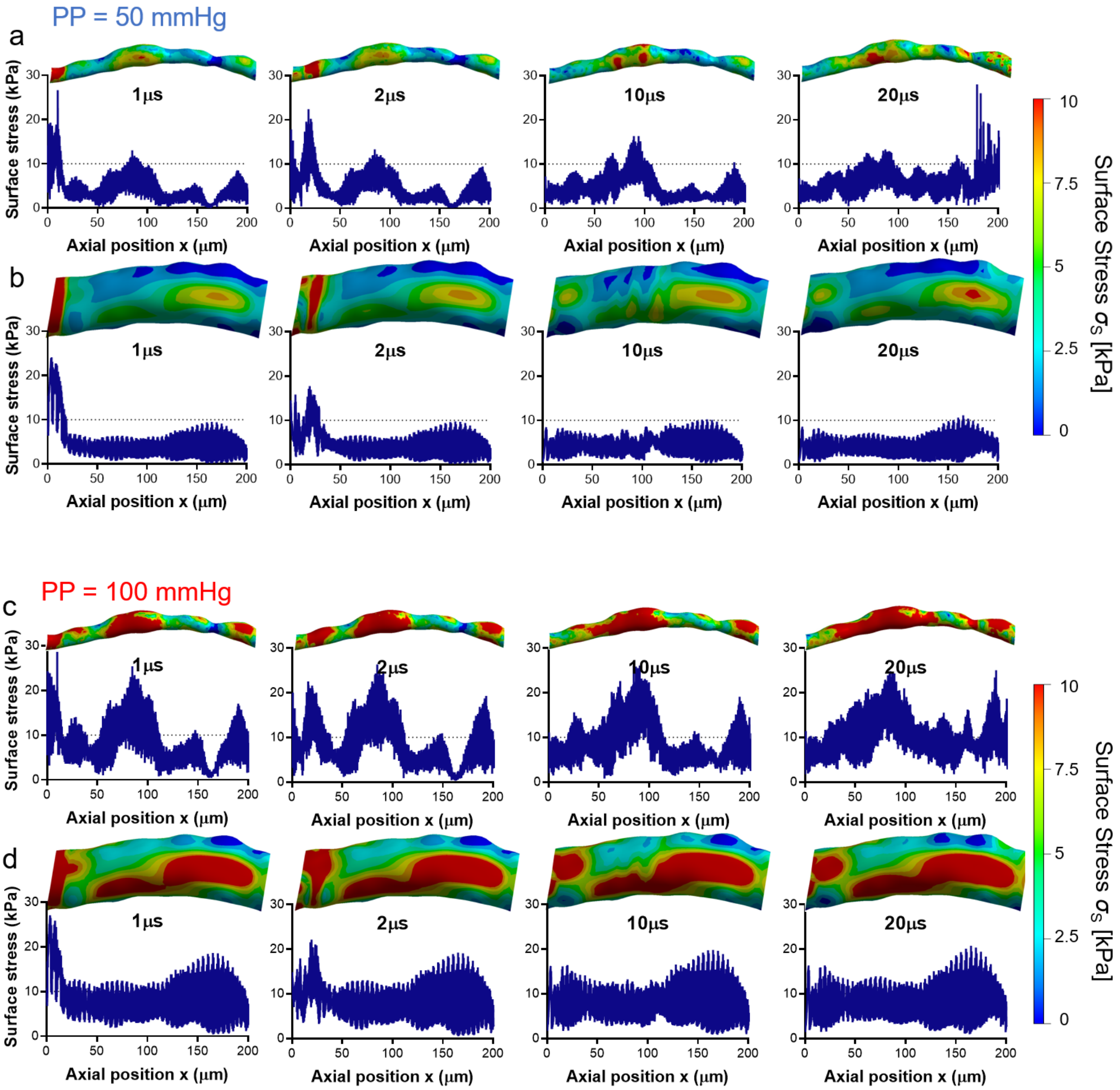

Figure S2. 3D simulation of dynamic vibratory stretching- and pulse pressure mediated contracting mechanotransduction in transverse vessels. a-b) The contour map of surface stress σ_S in transverse arteriole (a) and bone marrow artery (b) at $t = 1, 2, 10, 20$ μs after imposing a single mechanical pulsatile stretch under a pulse pressure of 50 mmHg. **c-d)** The contour map of surface stress σ_S in transverse arteriole (c) and bone marrow artery (d) at $t = 1, 2, 10, 20$ μs after imposing a single mechanical pulsatile stretch under a pulse pressure of 100 mmHg. With the presence of pulse pressure, the artery displays the fastest while the sinusoid displays the slowest in mechanotransduction. The impacted area was not differentiable from the pulse pressure-induced stress contour after $t = 10$ μs for both arteriole and artery.
